# *Fusarium verticillioides* and *Aspergillus flavus* Co-Occurrence Influences Plant and Fungal Transcriptional Profiles in Maize Kernels and In Vitro

**DOI:** 10.3390/toxins13100680

**Published:** 2021-09-24

**Authors:** Alessandra Lanubile, Paola Giorni, Terenzio Bertuzzi, Adriano Marocco, Paola Battilani

**Affiliations:** 1 Department of Sustainable Crop Production, Università Cattolica del Sacro Cuore, Via Emilia Parmense 84, 29122 Piacenza, Italy; alessandra.lanubile@unicatt.it (A.L.); paola.giorni@unicatt.it (P.G.); adriano.marocco@unicatt.it (A.M.); 2 Department of Animal Science, Food and Nutrition, Università Cattolica del Sacro Cuore, Via Emilia Parmense 84, 29122 Piacenza, Italy; terenzio.bertuzzi@unicatt.it

**Keywords:** pathogenesis-related genes, mycotoxin gene cluster, fumonisins, aflatoxins, temperature, competition, *Zea mays*

## Abstract

Climate change will increase the co-occurrence of *Fusarium verticillioides* and *Aspergillus flavus*, along with their mycotoxins, in European maize. In this study, the expression profiles of two *pathogenesis-related* (*PR*) genes and four mycotoxin biosynthetic genes, *FUM1* and *FUM13*, fumonisin pathway, and *aflR* and *aflD*, aflatoxin pathway, as well as mycotoxin production, were examined in kernels and in artificial medium after a single inoculation with *F. verticillioides* or *A. flavus* or with the two fungi in combination. Different temperature regimes (20, 25 and 30 °C) over a time-course of 21 days were also considered. In maize kernels, *PR* genes showed the strongest induction at 25 °C in the earlier days post inoculation (dpi)with both fungi inoculated singularly. A similar behaviour was maintained with fungi co-occurrence, but with enhanced defence response at 9 dpi under 20 °C. Regarding *FUM* genes, in the kernels inoculated with *F. verticillioides* the maximal transcript levels occurred at 6 dpi at 25 °C. At this temperature regime, expression values decreased with the co-occurrence of *A. flavus*, where the highest gene induction was detected at 20 °C. Similar results were observed in fungi grown in vitro, whilst *A. flavus* presence determined lower levels of expression along the entire time-course. As concerns *afl* genes, considering both *A. flavus* alone and in combination, the most elevated transcript accumulation occurred at 30 °C during all time-course both in infected kernels and in fungi grown in vitro. Regarding mycotoxin production, no significant differences were found among temperatures for kernel contamination, whereas in vitro the highest production was registered at 25 °C for aflatoxin B1 and at 20 °C for fumonisins in the case of single inoculation. In fungal co-occurrence, both mycotoxins resulted reduced at all the temperatures considered compared to the amount produced with single inoculation.

## 1. Introduction

Climate change (CC), with increasing temperature and CO_2_, different rain distribution and extreme events, impacts significantly on fungal populations, including mycotoxin-producing fungi. A shift between species causing *Fusarium* head blight on wheat in Europe, and consequently in the mycotoxin profile, was reported in Europe as a CC effect [1,2,3,4]. An unexpected outbreak of aflatoxin contamination in maize in Europe happened in 2003 [5] and this event is expected/confirmed to increase with CC [6,7,8,9]. Further, the prevalent mycotoxin in maize is variable, between years and growing areas, but the co-occurrence of different toxins is progressively highlighted [10,11,12,13,14], even in small size territories [15].

In this variable scenario, the attention is focused on *Aspergillus flavus* and *Fusarium verticillioides*, the key actors in maize kernels infection, aflatoxin (AF) and fumonisin (FB) producers, respectively [16,17]. Although these fungi can grow at a wide range of temperature and water activity (a_w_), the ideal growth condition for *A. flavus* is 30 °C [18], whereas *F. verticilliodies* is favoured by lower temperatures ranging between 20 and 25 °C [19]. However, in the event of co-occurrence of both fungi, on the same substrate, they can influence each other, causing a different result both in term of growth and in mycotoxin production [20]. Literature reports on fungi co-occurrence are very limited, but partially contradictory. In particular, some studies underlined the capacity of *F. verticillioides* to inhibit *A. flavus* both in growth and in aflatoxin B1 (AFB1) production [21,22], while, in some cases, *A. flavus* was reported to negatively influence *F. verticillioides* development (up to 44% reduction in growth) [20,22]. However, other studies found that when the two fungi were inoculated together in corn grains, all the mycotoxins were produced at higher amounts if compared with single fungus production [23]. In addition, the presence of weevils favoured the production of both fumonisins and aflatoxins, confirming the role of these insects as mechanical vectors for the toxigenic fungi [23].

Environmental parameters, in particular temperature and a_w_, can determine a different response both in plant capacity to face fungal attack and in fungal ability to growth and produce mycotoxins, especially in the case of fungal co-occurrence [20,22]. This reveals a lot of still undiscovered mechanisms involved in mycotoxigenic fungi co-occurrence and raises the necessity to better elucidate the highly dynamic and complex maize–*F. verticillioides*–*A. flavus* interactions.

The analysis of expression profiles of maize *pathogenesis-related* (*PR*) genes (*PR5* and *PRm3*) regarding defence mechanisms of plants [24,25] and fungal biosynthetic genes of the considered mycotoxins (*FUM1* and *FUM13* for fumonisin biosynthesis, and *aflR* and *aflD* for aflatoxin biosynthesis) represents a cornerstone approach in deeper understanding the way both maize and fungi interact. Data from previous research highlighted that both temperature and a_w_ regimes had a profound effect on gene expression of key mycotoxin biosynthetic genes, too [16]. In particular, relative expression of *FUM1* to *FUM13* genes was related and influenced by these two environmental factors for both FB1 and FB2 production [19]. Accordingly, significant and prolonged increase in water stress (a_w_ < 0.93) enhanced *FUM1* expression, while a lower stress (0.98–0.95 a_w_) did not affect transcript accumulation [19,26]. Similar trends were also observed for *aflR* and *aflD* genes [18].

Therefore, the aim of this study was to: (i) gain deeper insight into the cross-talk between maize and its main mycotoxigenic fungi (*F. verticillioides* and *A. flavus*), utilising a maize kernel inoculation assay; (ii) enrich the knowledge about *F. verticillioides* and *A. flavus* co-culture performance in in vitro experiments; (iii) quantify the influence of fungal co-occurrence on maize and fungal transcriptional profiles and mycotoxin production in different temperature regimes.

## 2. Results

### 2.1. Expression Analysis of Maize Pathogenesis-Related Genes in Infected Kernels

**Figure 1:**
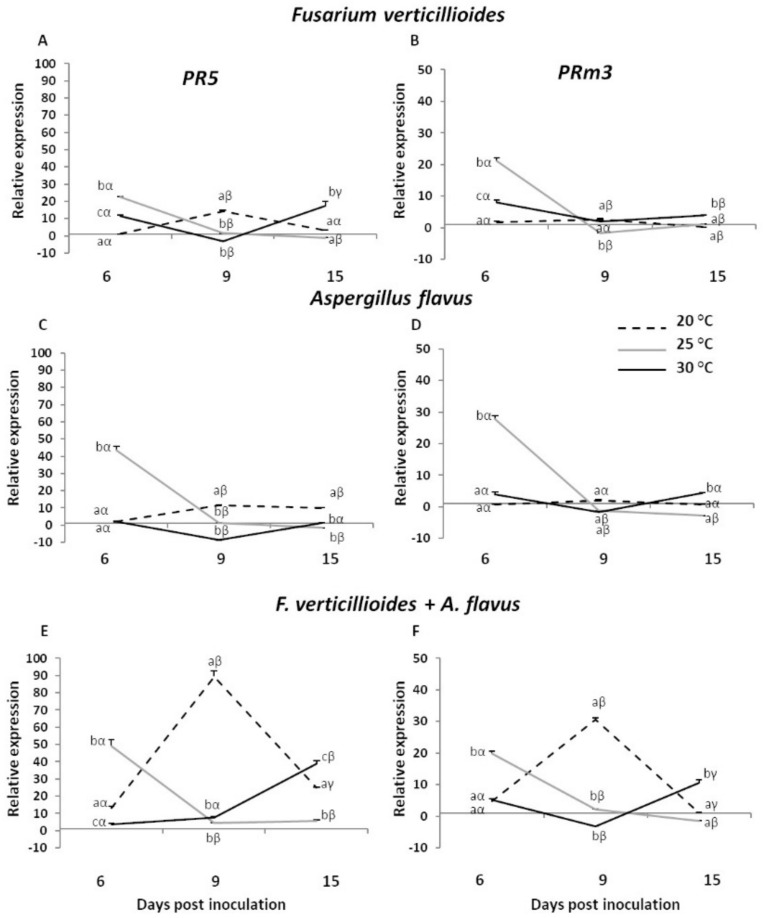
Relative expression of maize *PR5* and *PRm3* genes in kernels of B73 inbred line inoculated with *Fusarium verticillioides* (**A**,**B**), *Aspergillus flavus* (**C**,**D**) and *F. verticillioides* + *A. flavus* (**E**,**F**) at 6, 9 and 15 days post inoculation (dpi) under three different temperatures (20, 25 and 30 °C). Standard deviations (SD) of the mean are indicated by vertical bars (*n* = 3). The same letters over the line graphs state not significant differences among means of the three temperatures within each dpi (Latin letters) and the three dpi within each temperature (Greek letters), as resulting from Tukey’s honestly significant difference test (*p* ≤ 0.05).

When maize comes under pathogen attack, complex defence responses are initiated, including the activation of pathogenesis-related (PR) proteins. The expression profiles of two *PR* genes (*PR5* and *PRm3*) were measured by real-time RT-qPCR in the maize line B73 after inoculation with *F. verticillioides*, *A. flavus* and the two fungi in combination (Figure 1). The kernel fungal colonization bioassay, as described by Christensen et al. [27] and Battilani et al. [28], was performed on maize kernels under three different temperature regimes (20, 25 and 30 °C) to evaluate the possible effect of environmental factors during the host–pathogen interaction over a time-course of 15 days. The relative expression profiles were calculated as fold change (FC) of inoculated over mock-inoculated kernels (Table S1).

Similar expression profiles were reported for both *PR5* and *PRm3* genes considering the three variables examined in this study (fungal treatment, temperature and incubation time). More in detail, the expression of *PR5* gene appeared to be not notably affected 6 days post inoculation (dpi) with *F. verticillioides* inoculation at 20 °C (FC = 1.07), whereas a significant up-regulation was reported at 9 dpi (FC = 14.23), followed by a decrease at 15 dpi (FC = 3.27; Figure 1A). The gene *PR5* reached the highest level of induction at 6 dpi under 25 °C (FC = 22.57), while in contrast a significant drastic decline was detected at the later times of incubation. At 30 °C, *F. verticillioides* triggered an induction of about 12-fold at 6 dpi. A weak down-regulation was reported at 9 dpi, whereas at 15 dpi the gene was again significantly up-regulated (FC = 17.46). Comparable values of expression were detected for the *PRm3* gene, where it significantly peaked at 6 dpi under 25 °C (FC = 21.09; Figure 1B), whereas fewer striking differences were observed for the other dpi and temperatures.

As for *F. verticillioides*, *A. flavus* caused the strongest up-regulation at 6 dpi under 25 °C, even more marked compared to *Fusarium*, with FC values of about 44 and 28 for *PR5* and *PRm3*, respectively, followed by a drastic reduction at the later dpi (Figure 1C,D). At 20 °C, a peak of expression was reported for both genes at 9 dpi, albeit FC values resulting significantly different from the other two temperatures only for *PR5* gene (FC = 11.46). *PR* genes were significantly down-regulated at 30 °C 9 days after *A. flavus* inoculation showing expression levels of −8.60 and −1.70-fold for *PR5* and *PRm3*, respectively, while in contrast higher FC values once again were restored at 15 dpi.

Interestingly, when *F. verticillioides* and *A. flavus* were co-inoculated, an enhanced kernel response to fungal attack was observed and this was notably evident at 20 °C after 9 dpi, where a peak of expression of about 90- and 31-fold was reached by *PR5* and *PRm3*, respectively (Figure 1E,F). Similar expression profiles were detected for both genes at 25 °C, comparable to those reported for the two fungi inoculated individually, with the maximum expression observed at 6 dpi (FC = 49.1 and 19.86 for *PR5* and *PRm3*, respectively). At 30 °C the most striking significant differences resulted at 15 dpi, where maize kernels exacerbated their extreme attempt to fight against fungal attack triggering a strong induction of *PR5* and *PRm3* genes up to about 39- and 11-fold.

### 2.2. Expression Analysis of Fusarium verticillioides and Aspergillus flavus Mycotoxin Genes in Infected Kernels

**Figure 2:**
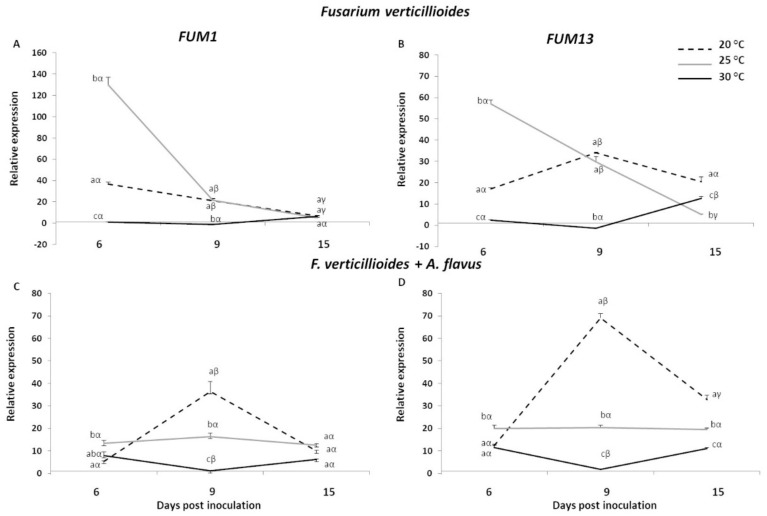
Relative expression of *Fusarium verticillioides FUM1* and *FUM13* genes in kernels of B73 inbred line inoculated with *F. verticillioides* (**A**,**B**) and *F. verticillioides* + *A. flavus* (**C**,**D**) at 6, 9 and 15 days post inoculation (dpi) under three different temperatures (20, 25 and 30 °C). Standard deviations (SD) of the mean are indicated by vertical bars (*n* = 3). The same letters over the line graphs state not significant differences among means of the three temperatures within each dpi (Latin letters) and the three dpi within each temperature (Greek letters), as resulting from Tukey’s honestly significant difference test (*p* ≤ 0.05).

**Figure 3:**
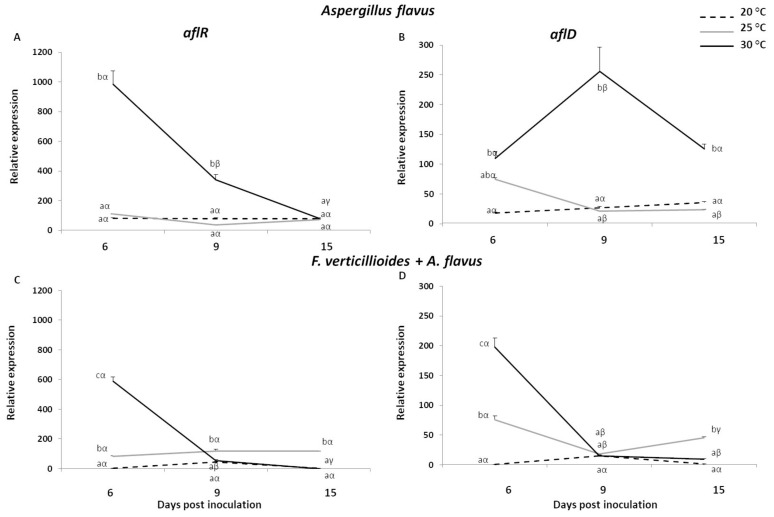
Relative expression of *Aspergillus flavus aflR* and *aflD* genes in kernels of B73 inbred line inoculated with *A. flavus* (**A**,**B**) and *F. verticillioides* + *A. flavus* (**C**,**D**) at 6, 9 and 15 days post inoculation (dpi) under three different temperatures (20, 25 and 30 °C). Standard deviations (SD) of the mean are indicated by vertical bars (*n* = 3). The same letters over the line graphs state not significant differences among means of the three temperatures within each dpi (Latin letters) and the three dpi within each temperature (Greek letters), as resulting from Tukey’s honestly significant difference test (*p* ≤ 0.05).

The biosynthetic gene clusters involved in the production of key mycotoxins such as fumonisins and aflatoxins have been largely unravelled [29,30,31]. The activities of four important genes involved in the mycotoxin production and regulation, *FUM1* and *FUM13*, of the fumonisin pathway, and *aflR* and *aflD*, of the aflatoxin pathway, were examined in this study (Figure 2 and Figure 3, Table S1). As for *PR* genes, the expression profiles of the four fungal genes were evaluated in kernels inoculated with the single fungus or co-inoculated, considering the same temperature regimes and times post-inoculation.

Regarding *FUM* genes, in the kernels inoculated with *F. verticillioides* the maximal transcript levels occurred at 6 dpi under 25 °C with FC values of 129.76 and 56.99 for *FUM1* and *FUM13*, respectively, followed by a marked decrease thereafter. The gene *FUM1* showed the same expression profile at 20 °C, albeit a lower induction of about three times was observed at 6 dpi (Figure 2A). Conversely, the gene *FUM13* peaked at 9 dpi under 20 °C (FC = 34.06), whereas FC values were significantly reduced at 15 dpi (Figure 2B). Transcript levels of both genes were not affected or even down-regulated at 30 °C 6 and 9 days after *F. verticillioides* inoculation, whereas a slight recovery was observed at the later time-point.

Interestingly, the co-occurrence of *A. flavus* negatively influenced the level of expression of *FUM* genes under 25 °C, to a greater extent for *FUM1* at 6 dpi, where we assisted to a reduction of about 9-fold (Figure 2C). In contrast, the highest gene induction was detected under 20 °C at 9 dpi with FC values of 36.44 and 69.16 for *FUM1* and *FUM13*, respectively (Figure 2C,D). Similar trends of expression were measured at 30 °C for both genes, albeit compared to *F. verticillioides* inoculated alone, where a stronger up-regulation of 7.88- and 11.44-fold was observed at 6 dpi for *FUM1* and *FUM13*, respectively.

Regarding *afl* genes, considering *A. flavus* alone, enhanced up-regulation levels were reached at 30 °C for both *aflR* and *aflD* genes during all time-course (Figure 3A,B). *AflR* showed the highest expression values with a remarkable 985.81-fold induction at 6 dpi. This value significantly declined of about three and twelve times at 9 and 15 dpi, respectively, where for the latter one, similar levels of expression were observed compared to the temperature regimes of 20 and 25 °C (Figure 3A). On the other hand, *aflD* gene showed a three-days-delayed peak of expression at 30 °C with a FC value of 256.00 (Figure 3B). No significant variation was displayed by both genes at 20 and 25 °C for all the time-points analysed, except *aflD* gene under 25 °C that resulted significantly up-regulated only at 6 dpi compared to 9 and 15 dpi.

Intriguingly, when fungi were co-inoculated in maize kernels, a minor impact was observed on the expression of *A. flavus* genes, where the highest induction remained at 30 °C (Figure 3C,D). Albeit with slightly lower FC values, *aflR* maintained the same trend observed with *A. flavus* alone, and the maximal transcript accumulation was measured at 6 dpi (FC = 586.96) followed by a drop thereafter. A similar behaviour was displayed by the gene *aflD*, where the greatest expression still occurred at 6 dpi (FC = 197.51), even higher compared to the fungus inoculated individually (FC = 109.81). Further, at 25 °C the presence of *F. verticillioides* apparently did not strongly impact *A. flavus* genes. *AflR* transcript accumulation remained constant throughout the time-course with an average FC of about 105, whereas *aflD* showed the same trend reported for the kernels inoculated with *A. flavus* individually.

As expected, at 20 °C gene induction was reduced for both *aflR* and *aflD*, confirming how *A. flavus* resulted more compromised at this temperature regime.

### 2.3. Mycotoxins Production in Infected Kernels

**Figure 4:**
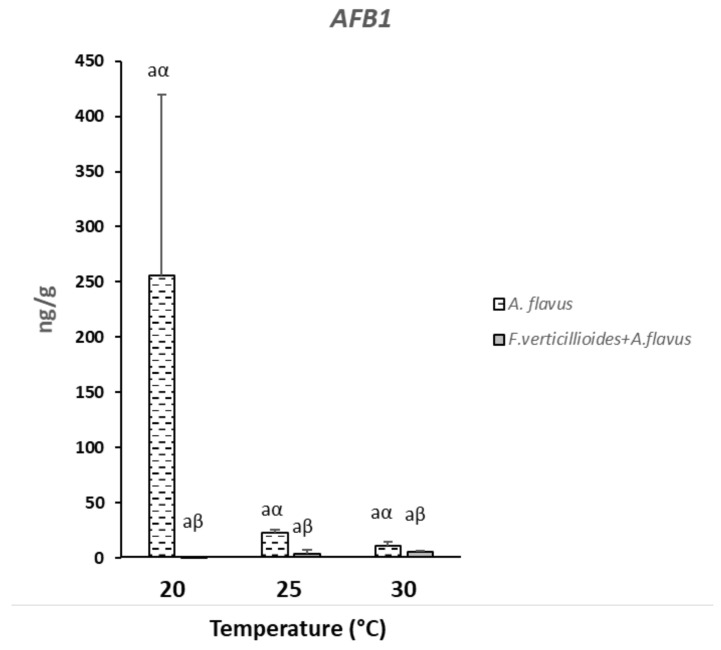
Aflatoxin B1 (AFB1) production in the case of single inoculum with *Aspergillus flavus* or in the case of inoculum with both *A. flavus* and *F. verticillioides* at the three different temperatures considered in the study (20, 25 and 30 °C) after 21 days of incubation. Standard deviations (SD) of the mean are indicated by vertical bars (*n* = 3). The same letters over the bars state not significant differences among means of the three temperatures within each inoculation condition (Latin letters) and the two theses within each temperature (Greek letters), as resulting from Tukey’s honestly significant difference test (*p* ≤ 0.05).

Maize kernels resulted to have significant different contamination regarding AFB1 among the considered inoculation conditions (*p* ≤ 0.01) (Tables S1 and S2). In particular, kernels inoculated with only *A. flavus* resulted to have a mean contamination almost 30 times higher than kernels where both *A. flavus* and *F. verticillioides* were present (96.8 vs. 3.3 ng/g AFB1). In the case of mock, AFB1 was found only in traces. No significant differences were found among all the temperatures considered (Figure 4). When both *A. flavus* and *F. verticillioides* were inoculated together, small amounts of AFB1 were found at all the tested temperatures (Figure 4).

Unfortunately, in all considered inoculation conditions and temperatures, FBs were never detected. This was probably due to the initial low a_w_ level of kernels.

### 2.4. Expression Analysis of Mycotoxin Genes in Fusarium verticillioides and Aspergillus flavus Grown In Vitro

**Figure 5:**
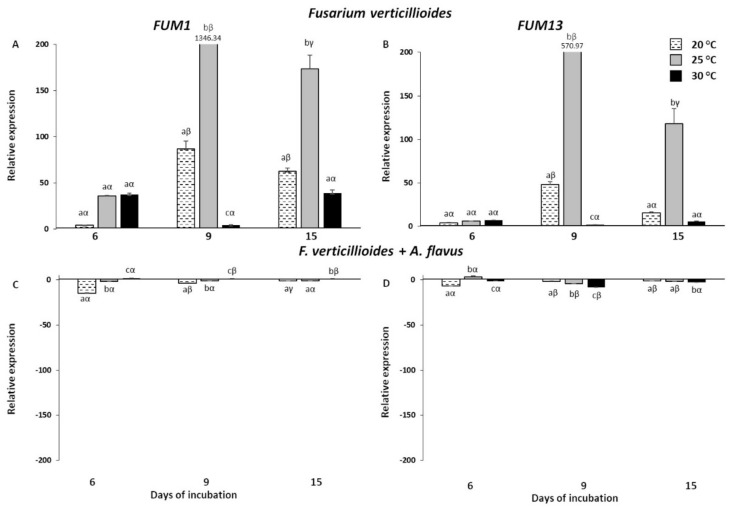
Relative expression of *Fusarium verticillioides FUM1* and *FUM13* genes in *F. verticillioides* (**A**,**B**) and *F. verticillioides* + *A. flavus* (**C**,**D**) grown on liquid medium at 6, 9 and 15 days of incubation under three different temperatures (20, 25 and 30 °C). Standard deviations (SD) of the mean are indicated by vertical bars (*n* = 3). The same letters over the histograms state not significant differences among means of the three temperatures within each dpi (Latin letters) and the three dpi within each temperature (Greek letters), as resulting from Tukey’s honestly significant difference test (*p* ≤ 0.05).

**Figure 6:**
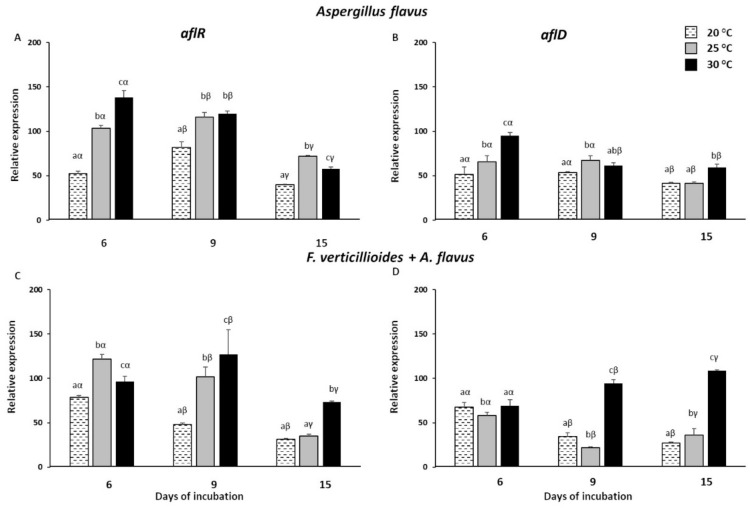
Relative expression of *Aspergillus flavus aflR* and *aflD* genes in *A. flavus* (**A**,**B**) and *F. verticillioides* + *A. flavus* (**C**,**D**) grown on liquid medium at 6, 9 and 15 days of incubation under three different temperatures (20, 25 and 30 °C). Standard deviations (SD) of the mean are indicated by vertical bars (*n* = 3). The same letters over the histograms state not significant differences among means of the three temperatures within each dpi (Latin letters) and the three dpi within each temperature (Greek letters), as resulting from Tukey’s honestly significant difference test (*p* ≤ 0.05).

The four mycotoxin biosynthetic genes, FUM1, FUM13, aflR and aflD, were also tested in *F. verticillioides* and *A. flavus* grown in liquid medium considering the same temperature regimes/days of incubation (doi) mentioned before (Figure 5 and Figure 6, Table S1).

Concerning FUM genes, *F. verticillioides* showed the highest transcript accumulation at 25 °C, especially at 9 doi, with an induction of 1346.34 and 570.97 for FUM1 and FUM13, respectively (Figure 5A,B). This was in line with the results obtained in inoculated kernels, though we assisted to a temporal shift of three days. Dramatically lower levels of expression were measured at the other two temperatures during the entire time-course, more pronounced at 30 °C, as already reported in the inoculated kernels.

Notably, the co-occurrence of A. flavus greatly inhibited the FC values of both genes for all the conditions considered (Figure 5C,D). This trend was more marked for the gene expression in *F. verticillioides* grown in vitro compared to the results obtained through the in vivo bioassay previously described, revealing how the different growth substrates (kernels vs. artificial liquid medium) can heavily influence fungal behaviour.

As regards afl genes, A. flavus exhibited the strongest up-regulation at 30 °C at 6 doi with FC values of 137.98 and 94.84 for aflR and aflD, respectively (Figure 6A,B), followed by a decline in the later doi. This finding supported the previous analysis carried out in the kernels, even though the differences among the three temperature regimes were less marked in vitro, where an induction of aflR and aflD at 20 and 25 °C was observed too.

Similar trends were found with the co-occurrence of *F. verticillioides*, where the expression of both genes did not look to be negatively influenced (Figure 6C,D). The optimum of temperature was once again confirmed at 30 °C, where the highest levels of expression were measured at almost all doi. However, transcripts accumulated for both afl genes at 20 and 25 °C as well, in line with the results obtained in inoculated kernels.

### 2.5. Mycotoxins Production by Fusarium verticillioides and Aspergillus flavus Grown In Vitro

Fumonisin and aflatoxin production were considered separately for each fungus both in the case of single fungus and in the case of co-occurrence of the two fungi.

**Figure 7:**
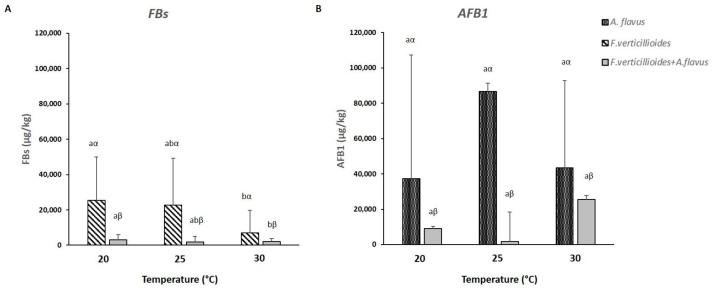
Fumonisin B1 + B2 (FBs) production in the case of single inoculum with *Fusarium verticillioides* alone or with *Aspergillus flavus* (**A**) and aflatoxin B1 (AFB1) production in the case of single inoculum with *Aspergillus flavus* alone or with *Fusarium verticillioides* (**B**) at the three different temperatures considered in the study (20, 25 and 30 °C) after 21 days of incubation. Standard deviations (SD) of the mean are indicated by vertical bars (*n* = 3). The same letters over the bars state not significant differences among means of the three temperatures within each inoculation condition (Latin letters) and the two theses within each temperature (Greek letters), as resulting from Tukey’s honestly significant difference test (*p* ≤ 0.05).

Regarding *F. verticillioides*, FB production was higher in the case of inoculum with only *F. verticillioides* in comparison with inoculation with both *F. verticillioides* and *A. flavus* (Tables S1 and S3); however, temperature resulted to have an important role in the case of FB production. In fact, in both cases, the highest production was found at 20 °C and the lowest at 30 °C (Figure 7A). Even statistically, differences among the different temperatures considered resulted to be significant (*p* ≤ 0.05) (Table S3).

Similarly, the highest AFB1 production was registered in the case of *A. flavus* inoculated alone with the maximum concentration at 25 °C (Figure 7B), while in the case of co-occurrence with *F. verticillioides*, AFB1 production resulted lower at all the temperatures considered. In particular, in the case of co-inoculation with both fungi, the highest production of AFB1 was registered at 30 °C (Figure 7B). From a statistical point of view, significant differences in AFB1 were found only between the production occurred with only A. flavus and with both *A. flavus* and *F. verticillioides* (*p* ≤ 0.05) while temperatures resulted not significant for AFB1 contamination (Table S3).

## 3. Discussion

Climate change impact on mycotoxin producing fungi and mycotoxin contamination in maize was highlighted [15], with increased co-occurrence in feed and food [12,32,33,34]. As a consequence of the global warming scenario, wider areas in Europe will face an increased risk of *A. flavus* occurrence and AFB1 contamination [6]. Moreover, a shift in the *Fusarium* population prevalence from *F. graminearum* to *F. verticillioides* and concomitant fumonisin production was reported in a multi-year investigation by Vandicke et al. [35]. Therefore, due to the expected increase in the occurrence and co-occurrence of *F. verticillioides* and *A. flavus*, it is crucial to study their impact on maize grain contamination.

Although some of the mechanisms affecting disease resistance were previously evaluated in the pathosystems *F. verticillioides*–maize [25,36,37,38] and *A. flavus*–maize [39,40,41], this study for the first time broadens the use of gene expression to the combined *F. verticillioides*–*A. flavus* infection, in order to better elucidate how defence gene systems can be influenced by fungal co-occurrence.

In this experiment, *PR5* and *PRm3* genes showed a similar trend of expression both considering the individual and combined fungal inoculation. The strongest induction was observed at 25 °C in the earlier dpi with the single *F. verticillioides* and *A. flavus* inoculation. The same behaviour was maintained in co-occurrence of both fungi, but with enhanced defence response at 9 dpi under 20 °C. These results suggest how mild temperature regimes (20–25 °C) favoured the readiness of maize to face invader pathogens, enhanced in response to fungal co-occurrence, and confirm the role of *PR5* and *PRm3* genes as maize defence markers against mycotoxigenic fungi. Accordingly, previous works showed, by RNA in situ hybridisation, the accumulation of *pathogenesis-related protein maize seeds* (*PRms*) genes in kernel tissues of maize after infection by *F. verticillioides* and *A. flavus* [41]. Lanubile and co-workers [39] reported the up-regulation of four *PR* genes, including *PR5* and *PRm3*, in kernels from adult plants in response to the mycotoxin-producing fungi *F. proliferatum*, *F. subglutinans* and *A. flavus*, confirming their strategic role in the reaction to several ear rot fungi attack.

While a decrease of *PR5* and *PRm3* gene expression was reported at the late stage of inoculation for all treatments under 20 and 25 °C, an opposite trend was observed at 30 °C, in a more remarkable way after fungal co-occurrence. It could be speculated that under suboptimal temperature conditions, kernels needed a more prolonged time lapse to activate their defence mechanisms and counteract fungal attack. More in-depth analysis will be required to verify this hypothesis.

This study also improves the knowledge on the effects of fungal co-occurrence and environmental conditions on the expression of two fumonisin biosynthetic genes. A higher transcription of both *FUM1* and *FUM13* genes was observed at 25 °C in the earlier times of incubation in kernels inoculated with *F. verticillioides*. Similarly, the fungus grown on artificial medium showed the maximum activation of *FUM* genes under the same temperature at 9 and 15 dpi, indicating that 25 °C was the optimum condition for the gene expression of the *F. verticillioides* strain used in this study, irrespective of the other abiotic factors considered. In fact, it is important to underline that a_w_ level of the substrates considered were very different being 0.73 for maize kernels and 0.97 for the artificial medium. The importance of temperature on gene expression was also pointed out by Marìn et al. [42], which reported an enhanced *FUM1* transcription at 20 °C under increasing water solute potentials ranging from 0.7 to 7.0 MPa. Additionally, Lazzaro et al. [43] focused on the genes *FUM2* and *FUM21* and found that temperature significantly influenced only the expression of *FUM21* at 25 °C, whereas a_w_ did not significantly affect gene expression. Medina and co-workers [19] examined in vitro the effect of temperature (20–35 °C) and a_w_ (0.93–0.995 a_w_) on *F. verticillioides* growth and fumonisin production and the expression of nine genes of the *FUM* cluster, including *FUM1* and *FUM13*. Data showed an increased transcript accumulation for *FUM1* at 20 °C and 0.95 a_w_, and *FUM13* at 25 °C and 0.93 a_w_, indicative of the effect of drought × temperature stress [19], partially in disagreement with the other reports.

Interestingly, when fungi co-occurred in kernels, *A. flavus* was disadvantaged at low temperatures (20 °C) and resulted less competitive than *F. verticillioides*, favoured by this temperature regime. Whereas, on one hand, in kernels the presence of *A. flavus* adversely affected *FUM1* and *FUM13* gene expression at 25 °C, the influence of temperature on transcript amount in fungi grown in vitro was less marked. Indeed, a general down-regulation of both genes was found for almost all conditions tested (temperature and days of incubation) in in vitro conditions. A previous work reported that *FUM* gene expression was enhanced during the milk stage (R3) compared to the other maize maturity stages [44]. In the present study, *FUM* gene expression was tested at hard dough/dent stages (R4-5), and this contribute to explain the partially in agreement findings. Moreover, host genetics and the employment of a susceptible line, such as B73, could have played a critical role in the fungal actions and subsequent activation of the *FUM* biosynthetic pathway. Further experiments focused on these variables will clarify our findings more accurately.

Regarding *afl* genes, boosted levels of expression were found for both *aflR* and *aflD* genes at 30 °C over the time-course of 15 days in kernels inoculated with *A. flavus* alone. The *aflR* and *aflD* peaked at 6 and 9 dpi, respectively. *AflD* is an early gene encoding a reductase responsible for the conversion of norsolorinic acid to averantin [45,46,47]. The temporal shift of three days observed in this study analysing *aflR* and *aflD* transcriptional profiles could be due to the role of *aflR* that, being a transcription activator of the aflatoxin biosynthetic pathway, was earlier induced, and showed a higher magnitude of activation in comparison to *aflD*. This trend was partially confirmed by the in vitro assay, where significant enhanced transcripts were detected at 30 °C for almost all doi, albeit the most striking differences were displayed at 6 doi. Previous studies carried out on *A. flavus* grown on artificial media have reported the effect of some environmental factors on the up- or down-regulation of several aflatoxin genes. RNASeq analysis outlined that most of the genes in the aflatoxin cluster were highly upregulated at 30 °C, while not expressed at 37 °C, with *aflD* resulting as the most induced gene [48]. Similarly, from Gallo et al.’s [49] results, 28 °C was identified as the key temperature, compared to 20 and 37 °C, able to activate the most elevated levels of structural (*aflD* and *aflO*) and regulatory (*aflR* and *aflS*) gene expression in *A. flavus* grown on almond medium. Findings from both studies support the same ideal temperature ascertained in our analyses and provide evidence of both *aflR* and *aflD* co-regulated activities.

Remarkably, co-presence of *A. flavus* and *F. verticillioides* did not affect the magnitude of *afl* transcript accumulation and the optimum temperature condition was confirmed at 30 °C. This was notably evident at the earlier dpi in the co-inoculated kernels, whereas temperature impact was less pronounced in fungi co-cultured in vitro, where significantly higher *aflD* and *aflR* induction occurred at 30 °C mainly during the late times of incubation (9 and 15 doi). Few examples in literature focusing on the interaction of mycotoxigenic fungal strains are available and most of them are based on the study of mycelium growth and toxin production [17,20,22]. For this reason, the comparison with this work is quite strenuous, since in our case the analysis of gene expression was employed as cornerstone approach in studying fungal co-occurrence. Recently, Camardo Leggieri et al. [22] reported a reciprocal influence when *A. flavus* and *F. verticillioides* were co-inoculated in plate, though the effect of *A. flavus* on *F. verticillioides* was stronger, determining a reduction of 44% in colony diameter versus a 10% of decrease determined by *F. verticillioides* on *A. flavus*. Likewise, the results from in vitro experiments by Chen et al. [17] showed that the growth rate of *A. flavus* and *F. verticillioides*, in dual or mixed culture, was distinctly reduced by 10% and 15%, respectively, confirming a slightly stronger impact of *A. flavus* on *F. verticillioides* compared to the opposite. In the same study, an opposite trend was observed from in planta experiments carried out in maize cobs, where the pre-inoculation of *A. flavus* did not affect *F. verticillioides* and its resulting fumonisin production. The different doi considered in vitro (4 days) and in planta (7 days) experiments could have influenced the contrasting outcomes. Furthermore, a plant-mediated defence response induced by the pre-inoculation with *A. flavus* could have inhibited *F. verticillioides* colonisation. These findings are in line with previous investigations that reported the dominance of *F. verticillioides* on *A. flavus*, *Penicillum* spp. and *F. graminearum* under field conditions [42,50,51]. However, in vitro and in vivo results about species dominance are difficult to set side by side, because strain and/or environment-specific factors should be taken in account.

Regarding mycotoxin production, similar results were obtained in this study on kernels and artificial medium. In general, mycotoxin production by single fungal inoculation were always higher than with co-occurrence of *A. flavus* and *F. verticillioides*. This was also found in a previous in vitro study where, similarly, both fungi were tested singularly and in co-presence [17]. However, in a study conducted on maize ears in field, this was confirmed for *F. verticillioides* while the highest production of AFB1 was recorded in the case of fungal co-occurrence [20], but the strong impact of weather conditions resulted in important differences between growing years.

In this study, no significant differences in AFB1 production were found among tested temperatures; on the opposite, maximum FB production in vitro was reported at 20 °C and 25 °C, reported as optimal for both mycotoxins by these two considered fungi [22]. FBs were never detected in kernels; this was probably due to the low a_w_ level of kernels (0.76 a_w_) that also affected AFB1 production, resulted very low. Optimal a_w_ values for AFB1 production are 0.95–0.99 [19], or lower based on in vivo studies [52], while values 0.98–0.99 are optimal for FBs [18]. Many papers indicate 0.85 a_w_ as the lowest level to have AFB1 production by *A. flavus* in in vitro conditions; however, on maize kernels, this fungus seems able to extend this ability even at lower a_w_ levels [53]. A previous study underlined the possible different behaviour of fungi in the case of in vivo trials especially considering the possible relevant influence of conducive temperatures [52].

In this work, different times of sampling were considered for gene expression (6, 9 and 15 dpi/doi) and mycotoxin production (21 dpi/doi) analysis. This choice was due in account of kernel viability, as already reported by Battilani et al. [28]. In contrast, early stages of incubation do not allow to measure and discern properly FBs and AFB1 synthesis, particularly for the kernel inoculation assay.

In maize kernels, the expression of *aflR* and *aflD* genes decreased under 30 °C at 15 dpi for *A. flavus* both inoculated alone or with *F. verticillioides*. This could explain the reduced production of AFB1 observed under the same temperature regime, compared to lower temperatures. Moreover, considering only *A. flavus* inoculation, at 20 °C gene expression remained always unvaried during all the time-course with a slight increase at 15 dpi that could justify the higher, even if not significant, AFB1 synthesis at this temperature.

Regarding aflatoxin production in vitro, when *A. flavus* was alone, *aflR* and *aflD* transcripts tended to be lower at 15 doi for all temperatures, whose differences looked flattening in line with the AFB1 levels. Interestingly, during co-occurrence the expression of both genes was enhanced at 15 doi under 30 °C, even higher than with single *A. flavus*, supporting the higher production of AFB1 at this temperature. Previous studies demonstrated that environmental parameters such as temperature or a_w_ can produce significant changes in the transcriptome of *A. flavus* both in vitro and in maize [54]. Additionally, Medina et al. [55] reported the significant up-regulation of both *aflR* and *aflD* genes with an increase in AFB1 production only at a certain temperature (37 °C), CO_2_ level (650/1000 ppm) and water stress conditions (0.95/0.92 a_w_). In a recent study, Baazeem et al. [56] found a parallel trend between *aflR* gene expression and AFB1 production by *A. flavus* grown on pistachio nut-based media at 35 °C, CO_2_ at 1000 ppm, and 0.98 a_w_, but not for the *aflD* gene.

As regards fumonisins and *FUM* gene expression in vitro, results were more aligned and 20–25 °C were confirmed to be the optimal temperatures when *F. verticillioides* was grown alone. With the two fungi in combination, a significant reduction of FBs was described at all temperatures, behaviour consistent with the lower levels of expression measured for *FUM1* and *FUM13* along the entire time-course. Previously, Lopez-Errasquín et al. [57] suggested a linear relationship between *FUM1* and *FUM19* expression and fumonisin synthesis for *F. verticillioides* incubated at 20 °C for 14 days. A positive correlation was also found between *FUM1* and *FUM21* transcripts and FB production at different a_w_ and temperatures by Lazzaro et al. [43]. Conversely, Van Zyl and co-workers [58] reported a lack of correlation between these two parameters in maize kernels. Once again, these findings underline how different experimental conditions (in vitro vs. in planta) can heavily influence fungal transcriptome and the resulting toxin production and point out the highly complex and dynamic connection between genes and secondary metabolites.

In light of these infection experiments in kernels and in vitro, outcomes suggest that *F. verticillioides*–*A. flavus*–maize interactions depended on the temporal sequence of host infection establishment and fungal incubation as well as on the different temperature regimes. Even though much more data is required to enable an improved understanding of fungal and maize ecophysiology, all together these findings represent pivotal steps for elucidating complex fungal competition and better managing and controlling pathogen co-occurrence under CC scenario.

## 4. Materials and Methods

### 4.1. Plant Material, Fungal Strains and Inoculation Assay

The maize inbred line B73 was used as a susceptible genotype to both *F. verticillioides* and *A. flavus* infection [28,59]. Maize kernels were chosen of similar shape and size without visible defects. Water activity of kernels was measured before starting the experiment using AquaLab Pre (Meter Food, Pullman, WA, USA) resulting in 0.76 a_w_. Kernels were prepared following the methodology described in Battilani et al. [28]. Briefly, after wounding them to facilitate fungal infection and surface disinfection, groups of 4 kernels were placed in sterile 10 mL falcon tubes.

One strain of *A. flavus* (ITEM 8069) and one strain of *F. verticillioides* (ITEM 10027) able to produce, respectively, AFBs (B1 and B2) and FBs (B1, B2, B3) and stored in the official fungal collection of the Institute of Sciences of Food Production of the National Research Council (ISPA-CNR) in Bari were used for inoculum preparation. The strains were singularly inoculated on Petri dishes (Ø 9 cm) with Potato Dextrose Agar (PDA, Biolife, Milano, Italy) and incubated at 25 °C for 7 days (12 h light/12 h dark photoperiod). At the end of incubation, fungal colonies were washed with 10 mL of sterile distilled water. The obtained conidial suspension of each fungus was adjusted to a concentration of 10^6^ spores/mL using a Burker chamber for spore count and 200 µL inoculum used to inoculate maize grains. Four inoculation conditions were considered: kernels inoculated with *A. flavus*, kernels inoculated with *F. verticillioides*, kernels inoculated with both fungi and kernels inoculated with sterile water (mock).

Samples of the different inoculation conditions were put in falcon tubes, capped and mixed by vortex for 15 s, then incubated at 3 different temperatures (20, 25 and 30 °C) and sampled at 3 sampling times (6, 9 and 15 days of incubation). The trial was managed in triplicate.

The same fungal strains were used also for an in vitro assay on liquid medium. Conical flasks containing 50 mL of Potato Dextrose Broth (PDB) were prepared and each one inoculated with one 0.25 cm^2^ plug taken from each fungal colony grown on PDA. Three inoculation conditions were considered: inoculum with only *A. flavus*, inoculum with only *F. verticillioides*, inoculum with both fungi (*A. flavus* and *F. verticillioides*).

Flasks were incubated at 3 different temperatures (20, 25 and 30 °C) and sampled after 6, 9 and 15 days. Each inoculation condition was managed in triplicate.

All samples were stored at −80 °C until analysis. Gene expression and mycotoxin quantification were managed.

### 4.2. RNA Isolation and Real-Time RT-qPCR Expression Analysis

Maize samples and fungal mycelia were ground under liquid nitrogen with a pestle and mortar, and total RNA extraction and purification were carried out according to [39,40]. The amount and the quality of the total RNA was verified by fluorometric assay (Quibit, Thermo Fisher Scientific Inc. Waltham, MA, USA) as well as by agarose gel electrophoresis. Real-time reverse transcription-quantitative PCR (RT-qPCR) experiments were performed on kernels and fungal mycelia collected at 6, 9 and 15 days post inoculation/days of incubation with the single fungus or in combination after incubation with different temperature regimes (20, 25 and 30 °C) using the FluoCycle™ II SYBR Green master mix (EuroClone S.p.a., Milan, Italy) and the CFX-96 device (Bio-Rad, Hercules, CA, USA). One µg of total RNA was used for cDNA synthesis using the High Capacity cDNA Reverse Transcription Kit (Thermo Fisher Scientific). Twenty ng of single strand cDNA determined by fluorometric assay (Qubit, Thermo Fisher Scientific) were used for real-time RT-qPCR. Relative RT-qPCR was performed under the following conditions: 95 °C for 3 min and 40 cycles at 95 °C 15 s, 55–60 °C for 30 s, followed by a melting curve analysis [39,40]. Three technical replicates (within each biological replicate) were employed for each tested sample and template-free negative controls. Gene-specific primers are listed in Table 1. Relative quantification was normalised to the reference housekeeping genes *β*-*actin* for maize, *FvCalmodulin* for *F. verticillioides* and *AfCalmodulin* for *A. flavus*. Fold changes (FC) values in gene expression were calculated using the 2^−ΔΔCt^ method [60] and calibrated on the mock-inoculated kernels.

### 4.3. Mycotoxin Analyses

Analyses and standard preparations were performed according to the methods reported by Bertuzzi et al. [63] for AFB1 and by Pietri and Bertuzzi [64] for FBs. Kernels were treated with liquid nitrogen and milled using mortar and pestle. Fungal colonies from the in vitro experiment were separated by the liquid medium and both mycelium and broth were analysed separately.

AFB1 was extracted using acetone:water 7 + 3 *v*/*v* and purified trough immuno-affinity column (Easy-Extract Aflatoxin, R-Biopharm Rhône LTD, Glasgow, Scotland, United Kingdom); then, the mycotoxin was determined by a HPLC (High Performance Liquid Chromatography) instrument with fluorescence detector. Chromatographic separation was carried out on a Superspher RP-18 column (4 μm particle size, 125 × 4 mm i.d., Merck) at ambient temperature with a mobile phase water-methanol-acetonitrile (64 + 23 + 13, *v*/*v*/*v*). AFB1 were detected after post-column photochemical derivatisation (UVE, LCTech GmbH, Dorfen, Germany); the fluorimeter was set at 365 nm excitation and 440 nm emission wavelengths. The limit of detection (LOD) and the limit of quantification (LOQ) were 0.05 and 0.15 μg/kg, respectively.

Regarding FBs, after extraction with phosphate buffer and purification through immuno-affinity column (FumoniTest, VICAM, Milford, MA, USA), FBs were quantified by a HPLC-MS/MS (High Performance Liquid Chromatography coupled with mass spectrometer) system. In particular, FBs were separated on a Betasil RP-18 column (5 μm particle size, 150 × 2.1 mm, Thermo Fisher Scientific) with a mobile-phase gradient acetonitrile-water (both acidified with 0.2% formic acid) from 25:75 to 55:45 in 9 min, then isocratic for 3 min; gradient to 75:25 in 1 min and isocratic for 3 min (wash-step). The ionisation was carried out with an ESI interface (Thermo Fisher Scientific) in positive mode as follows: spray capillary voltage 4.5 kV, sheath and auxiliary gas 35 and 14 psi, respectively, temperature of the heated capillary 270 °C. For fragmentation of [M + H]+ ions (722 m/z for FB1 and 706 m/z for FB2), the argon collision pressure was set to 1.5 m Torr and the collision energy to 36 V. The selected fragment ions were: 704, 352, and 334 m/z for FB1, 688, 336, and 318 m/z for FB2. The LOD and the LOQ were 10 and 30 μg/kg, respectively.

For samples inoculated with both fungal species, AFB1 and FBs were extracted using an acetonitrile–methanol–water (25:25:50) solution considered suitable for both kind of mycotoxins. After extraction, samples were destined to AFB1 or FBs analysis as already described.

### 4.4. Statistical Analyses

For gene expression analyses, standard deviations of the means were calculated on three biological replicates. One-factor analysis of variance (ANOVA), followed by Tukey’s HSD test (*p* ≤ 0.05), was performed on the observed means of FC gene expression values to set significant differences among temperatures (20, 25 and 30 °C) within each dpi and among dpi (6, 9 and 15) within each temperature for each fungal treatment (*F. verticillioides*, *A. flavus* and *F. verticillioides* + *A. flavus*).

Regarding mycotoxins, all values were ln transformed before statistical analysis [65] and data obtained were subjected to univariate analysis of variance (ANOVA) using the generalised linear model (GLM) procedure and significant differences between means were confirmed using Tukey test (*p* ≤ 0.05).

The statistical package IBM SPSS statistics 25 (IBM Corp., Armonk, NY, USA) was used for data analysis.

## Figures and Tables

**Table 1:** Primer sequences for real-time RT-qPCR analyses.

Gene	Primer Forward (5′ → 3′)	Primer Reverse (5′ → 3′)	Source
*β*-*actin*	ATGGTCAAGGCCGGTTTCG	TCAGGATGCCTCTCTTGGCC	AY273142.1 [61]
*PR5*	GTCATCGACGGCTACAACCT	GGGCAGAAGGTGACTTGGTA	U82201.1 [39]
*PRm3*	GGCTCTACGCCTACGTCAAC	GATGGAGAGGAGCACCTTGA	S82314.1 [39]
*FvCalmodulin*	GATGGCCAGATTACCACCAA	CGCCATCATGGTAAGGAACT	HQ412321.1 [25]
*FUM1*	GAGCCGAGTCAGCAAGGATT	AGGGTTCGTGAGCCAAGGA	AF155773 [57]
*FUM13*	GCCTTTGGTCTTGTTCTCTCA	CGTCAATTATTGCCTCTTTCAA	AF155773 [62]
*AfCalmodulin*	GGCCGACTCTTTGACTGAAG	CATGTCCTGGAGTTCCGACT	XM_002374071.1 [39]
*aflR*	GAGCAAAGCACCCTGTCTTC	GTCGACTCGGCCAAGAAATC	XM_002379905 [46]
*aflD*	GCGCAAGTTCCACTTTGAGA	CCTTGGTCGCCCATATCAGT	AFLA_139390 [49]

## Data Availability

Data is contained within the article or Supplementary Materials.

## Supplementary Materials

The following are available online at https://www.mdpi.com/article/10.3390/toxins13100680/s1, Table S1: Sum of gene expression and mycotoxin mean data obtained in maize kernels and in vitro experiments. Table S2: Analysis of variance (ANOVA) of aflatoxin B1 (AFB1) contamination in maize kernels after 21 days of incubation in the different theses considered (mock, inoculation with *A. flavus*, inoculation with *A. flavus* and *F. verticillioides* at three different temperatures (20, 25 and 30 °C). Data refer to mean data; all experiments were conducted with three replicates; Table S3: Analysis of variance (ANOVA) of fumonisins B1 + B2 (FBs) and aflatoxin B1 (AFB1) contamination in artificial liquid medium (PDB) after 21 days of incubation in the different theses considered (inoculation with *F. verticillioides*, inoculation with *A. flavus*, inoculation with *A. flavus* and *F. verticillioides*) at three different temperatures (20, 25 and 30 °C).

## References

[1] Logrieco A.F., Moretti A. (2008). Between emerging and historical problems: An overview of the main toxigenic fungi and mycotoxin concerns in Europe. Mycotoxins: Detection Methods, Management, Public Health and Agricultural Trade.

[2] Nielsen L.K., Jensen J.D., Nielsen G.C., Jensen J.E., Spliid N.H., Thomsen I.K., Justesen A.F., Collinge D.B., Jorgensen L.N. (2011). Fusarium head blight of cereals in Denmark: Species complex and related mycotoxins. Phytopathology.

[3] Van der Lee T., Zhang H., van Diepeningen A., Waalwijk C. (2015). Biogeography of *Fusarium graminearum* species complex and chemotypes: A review. Food Addit. Contam. Part A.

[4] Valverde-Bogantes E., Bianchini A., Herr J.R., Rose D.J., Wegulo S.N., Hallen-Adams H.E. (2020). Recent population changes of Fusarium head blight pathogens: Drivers and implications. Can. J. Plant Pathol..

[5] Piva G., Battilani P., Pietri A., Barug D., Bhatnagar D., van Egmond H.P., van der Kamp J.W., van Osenbruggen W.A., Visconti A. (2006). Emerging issues in Southern Europe: Aflatoxins in Italy. The Mycotoxin Factbook. Food and Feed Topics.

[6] Battilani P., Toscano P., Van der Fels-Klerx H., Moretti A., Leggieri M.C., Brera C., Rortais A., Goumperis T., Robinson T. (2016). Aflatoxin B1 contamination in maize in Europe increases due to climate change. Sci. Rep..

[7] Dobolyi C., Sebok F., Varga J., Kocsube S., Szigeti G., Baranyi N., Szecsi A., Toth B., Varga M., Kriszt B. (2013). Occurrence of aflatoxin producing *Aspergillus flavus* isolates in maize kernel in Hungary. Acta Aliment..

[8] Levic J., Gosic-Dondo S., Ivanovic D., Stankovic S., Krnjaja V., Bocarov-Stancic A., Stepanic A. (2013). An outbreak of *Aspergillus* species in response to environmental conditions in Serbia. Pestic. Fitomed..

[9] Leggieri M.C., Toscano P., Battilani P. (2021). Predicted aflatoxin b1 increase in europe due to climate change: Actions and reactions at global level. Toxins.

[10] Krnjaja V., Levic J., Stankovic S., Petrovic T., Tomic Z., Mandic V., Bijelic Z. (2013). Moulds and mycotoxins in stored maize grains. Biotech. Anim. Husb..

[11] Smith M.C., Madec S., Coton E., Hymery N. (2016). Natural co-occurrence of mycotoxins in foods and feeds and their in vitro combined toxicological effects. Toxins.

[12] Ma R., Zhang L., Liu M., Su Y.T., Xie W.M., Zhang N.Y., Dai J.F., Wang Y., Rajput S.A., Qi D.S. (2018). Individual and combined occurrence of mycotoxins in feed ingredients and complete feeds in China. Toxins.

[13] Palumbo R., Crisci A., Venâncio A., Abrahantes J.C., Dorne J.-L., Battilani P., Toscano P. (2020). Occurrence and co-occurrence of mycotoxins in cereal-based feed and food. Microorganisms.

[14] Zhao L., Zhang L., Xu Z., Liu X., Chen L., Dai J., Karrow N.A., Sunet L. (2021). Occurrence of Aflatoxin B1, deoxynivalenol and zearalenone in feeds in China during 2018–2020. J. Anim. Sci. Biotechnol..

[15] Leggieri M.C., Lanubile A., Dall’Asta C., Pietri A., Battilani P. (2020). The impact of seasonal weather variation on mycotoxins: Maize crop in 2014 in northern Italy as a case study. World Mycotoxin J..

[16] Magan N., Medina A. (2016). Integrating gene expression, ecology and mycotoxin production by *Fusarium* and *Aspergillus* species in relation to interacting environmental factors. World Mycotoxin J..

[17] Chen X., Landschoot S., Detavernier C., De Saeger S., Rajkovic A., Audenaert K. (2021). Cross-talk between *Fusarium verticillioides* and *Aspergillus flavus* in vitro and in planta. Mycotoxin Res..

[18] Abdel-Hadi A., Schmidt-Heydt M., Parra R., Geisen R., Magan N. (2012). A systems approach to model the relationship between aflatoxin gene cluster expression, environmental factors, growth and toxin production by *Aspergillus flavus*. J. R. Soc. Interface.

[19] Medina A., Schmidt-Heydt M., Cárdenas-Chávez D.L., Parra R., Geisen R., Magan N. (2013). Integrating toxin gene expression, growth and fumonisin B1 and B2 production by a strain of *Fusarium verticillioides* under different environmental factors. J. R. Soc. Interface.

[20] Giorni P., Bertuzzi T., Battilani P. (2019). Impact of fungi co-occurrence on mycotoxin contamination in maize during the growing season. Front. Microbiol..

[21] Zummo N., Scott G.E. (1992). Interaction of *Fusarium verticillioides* and *Aspergillus flavus* on kernel infection and aflatoxin contamination in maize ears. Plant Dis..

[22] Leggieri M.C., Giorni P., Pietri A., Battilani P. (2019). *Aspergillus flavus* and *Fusarium verticillioides* interaction: Modeling the impact on mycotoxin production. Front. Microbiol..

[23] Ferreira-Castro F.L., Potenza M.R., Rocha L.O., Correa B. (2012). Interaction between toxigenic fungi and weevils in corn grain samples. Food Control.

[24] Majumdar R., Rajasekaran K., Sickler C., Lebar M., Musungu B.M., Fakhoury A.M., Payne G.A., Geisler M., Carter-Wientjes C., Wei Q. (2017). The pathogenesis-related maize seed (*PRms*) gene plays a role in resistance to *Aspergillus flavus* infection and aflatoxin contamination. Front. Plant Sci..

[25] Maschietto V., Lanubile A., De Leonardis S., Marocco A., Paciolla C. (2016). Constitutive expression of pathogenesis-related proteins and antioxydant enzyme activities triggers maize resistance towards *Fusarium verticillioides*. J. Plant Physiol..

[26] Jurado M., Marin P., Magan N., Gonzalez-Jaen M.T. (2008). Relationship between solute and matric potential stress, temperature, growth, and *FUM1* gene expression in two *Fusarium verticillioides* strains from Spain. Appl. Environ. Microbiol..

[27] Christensen S.A., Borrego E., Shim W., Isakeit T., Kolomiets M. (2012). Quantification of fungal colonization, sporogenesis, and production of mycotoxins using kernel bioassays. J. Vis. Exp..

[28] Battilani P., Lanubile A., Scala V., Reverberi M., Gregori R., Falavigna C., Dall’asta C., Park Y.S., Bennett J., Borrego E.J. (2018). Oxylipins from both pathogen and host antagonize jasmonic acid-mediated defence via the 9-lipoxygenase pathway in *Fusarium verticillioides* infection of maize. Mol. Plant Pathol..

[29] Proctor R.H., Plattner R.D., Desjardins A.E., Busman M., Butchko R.A.E. (2006). Fumonisin production in the maize pathogen *Fusarium verticillioides*: Genetic basis of naturally occurring chemical variation. J. Agric. Food Chem..

[30] Desjardins A.E., Proctor R.H. (2007). Molecular biology of *Fusarium* mycotoxins. Int. J. Food Microbiol..

[31] Caceres I., Al Khoury A., El Khoury R., Lorber S., Oswald I.P., El Khoury A., Atoui A., Puel O., Bailly J.-D. (2020). Aflatoxin biosynthesis and genetic regulation: A review. Toxins.

[32] Juroszek P., von Tiedemann A. (2013). Climatic changes and the potential future importance of maize diseases: A short review. J. Plant Dis. Prot..

[33] Medina A., Akbar A., Baazeem A., Rodriguez A., Magan N. (2017). Climate change, food security and mycotoxins: Do we know enough?. Fungal Biol. Rev..

[34] Logrieco A., Battilani P., Leggieri M.C., Jiang Y., Haesaert G., Lanubile A., Mahuku G., Mesterhazy A., Ortega-Beltran A., Pasti M. (2021). Perspectives on global mycotoxin issues and management from the MycoKey Maize Working Group. Plant Dis..

[35] Vandicke J., De Visschere K., Croubels S., De Saeger S., Audenaert K., Haesaert G. (2019). Mycotoxins in Flanders’ fields: Occurrence and correlations with *Fusarium* species in whole-plant harvested maize. Microorganisms.

[36] Lanubile A., Ferrarini A., Maschietto V., Delledonne M., Marocco A., Bellin D. (2014). Functional genomic analysis of constitutive and inducible defence responses to *Fusarium verticillioides* infection in maize genotypes with contrasting ear rot resistance. BMC Genom..

[37] Lanubile A., Maschietto V., Borrelli V.M., Stagnati L., Logrieco A.F., Marocco A. (2017). Molecular basis of resistance to Fusarium ear rot in maize. Front. Plant Sci..

[38] Lanubile A., Borrelli V.M., Soccio M., Giorni P., Stagnati L., Busconi M., Marocco A. (2021). Loss of *ZmLIPOXYGENASE4* decreases *Fusarium verticillioides* resistance in maize seedlings. Genes.

[39] Lanubile A., Maschietto V., De Leonardis S., Battilani P., Paciolla C., Marocco A. (2015). Defence responses to mycotoxin-producing fungi *Fusarium proliferatum*, *F. subglutinans*, and *Aspergillus flavus* in kernels of susceptible and resistant maize genotypes. Mol. Plant Microbe Interact..

[40] Lanubile A., Maschietto V., Battilani P., Marocco A. (2017). Infection with toxigenic and atoxigenic strains of *Aspergillus flavus* induces different transcriptional signatures in maize kernels. J. Plant Interact..

[41] Shu X., Livingston D.P., Franks R.G., Boston R.S., Woloshuk C.P., Payne G.A. (2015). Tissue-specific gene expression in maize seeds during colonization by *Aspergillus flavus* and *Fusarium verticillioides*. Mol. Plant Pathol..

[42] Marín S., Sanchis V., Ramos A. (1998). Environmental factors, in vitro interactions, and niche overlap between *Fusarium moniliforme*, *F. proliferatum*, and *F. graminearum*, *Aspergillus* and *Penicillium* species from maize. Mycol. Res..

[43] Lazzaro I., Susca A., Mulè G., Ritieni A., Ferracane R., Marocco A., Battilani P. (2012). Effects of temperature and water activity on *FUM2* and *FUM21* gene expression and fumonisin B production in *Fusarium verticillioides*. Eur. J. Plant Pathol..

[44] Rocha L.O., Reis G.M., Fontes L.C., Piacentini K.C., Barroso V.M., Reis T.A., Pereira A.A., Corrêa B. (2017). Association between *FUM* expression and fumonisin contamination in maize from silking to harvest. Crop Prot..

[45] Yu J., Chang P.K., Ehrlich K.C., Cary J.W., Bhatnagar D., Cleveland T.E., Payne G.A., Linz J.E., Woloshuk C.P., Bennett J.W. (2004). Clustered pathway genes in aflatoxin biosynthesis. Appl. Environ. Microbiol..

[46] Hua S.S., Beck J.J., Sarreal S.B.L., Gee W. (2014). The major volatile compound 2-phenylethanol from the biocontrol yeast, *Pichia anomala*, inhibits growth and expression of aflatoxin biosynthetic genes of *Aspergillus flavus*. Mycotoxin Res..

[47] Dooso Oloo R., Okoth S., Wachira P., Mutiga S., Ochieng P., Kago L., Nganga F., Domelevo Entfellner J.B., Ghimire S. (2019). Genetic profiling of *Aspergillus* isolates with varying aflatoxin production potential from different maize-growing regions of Kenya. Toxins.

[48] Yu J., Fedorova N.D., Montalbano B.G., Bhatnagar D., Cleveland T.E., Bennett J.W., Nierman W.C. (2011). Tight control of mycotoxin biosynthesis gene expression in *Aspergillus flavus* by temperature as revealed by RNA-Seq. FEMS Microbiol. Lett..

[49] Gallo A., Solfrizzo M., Epifani F., Panzarini G., Perrone G. (2016). Effect of temperature and water activity on gene expression and aflatoxin biosynthesis in *Aspergillus flavus* on almond medium. Int. J. Food Microbiol..

[50] Zorzete P., Castro R.S., Pozzi C.R., Israel A.L.M., Fonseca H., Yanaguibashi G., Corrêa B. (2008). Relative populations and toxin production by *Aspergillus flavus* and *Fusarium verticillioides* in artificially inoculated corn at various stages of development under field conditions. J. Sci. Food Agric..

[51] Picot A., Hourcade-Marcolla D., Barreau C., Pinson-Gadais L., Caron D., Richard-Forget F., Lannou C. (2012). Interactions between *Fusarium verticillioides* and *Fusarium graminearum* in maize ears and consequences for fungal development and mycotoxin accumulation. Plant Pathol..

[52] Giorni P., Bertuzzi T., Battilani P. (2016). Aflatoxin in maize, a multifaceted answer of *Aspergillus flavus* governed by weather, host-plant and competitor fungi. J. Cereal Sci..

[53] Garcia-Cela E., Kiaitsi E., Sulyok M., Krska R., Medina A., Damico I.P., Magan N. (2019). Influence of storage environment on maize grain: CO2 production, dry matter losses and aflatoxins contamination. Food Addit. Contam. Part A.

[54] Gilbert M.K., Medina A., Mack B.M., Lebar M., Rodriguez A., Bhatnagar D., Magan N., Obrian G., Payne G. (2018). Carbon dioxide mediates the response to temperature and water activity levels in *Aspergillus flavus* during infection of maize kernels. Toxins.

[55] Medina A., Rodriguez A., Magan N. (2015). Climate change and mycotoxigenic fungi: Impacts on mycotoxin production. Curr. Opin. Food Sci..

[56] Baazeem A., Rodriguez A., Medina A., Magan N. (2021). Impacts of climate change interacting abiotic factors on growth, *aflD* and *aflR* gene expression and aflatoxin B1 production by *Aspergillus flavus* strains in vitro and on pistachio nuts. Toxins.

[57] Lopez-Errasquín E., Vazquez C., Jimenez M., Gonzalez-Jaen M.T. (2007). Real-Time RT-PCR assay to quantify the expression of *FUM1* and *FUM19* genes from the fumonisin-producing *Fusarium verticillioides*. J. Microbiol. Methods.

[58] Van Zyl K., Rose L.J., Viljoen A. (2019). *Fusarium verticillioides FUM1* and *FUM19* gene expression in maize kernels during early infection. Physiol. Mol. Plant Pathol..

[59] Gao X., Brodhagen M., Isakeit T., Brown S.H., Göbel C., Betran J., Feussner I., Keller N.P., Kolomiets M.V. (2009). Inactivation of the lipoxygenase *ZmLOX3* increases susceptibility of maize to *Aspergillus* spp.. Mol. Plant Microbe Interact..

[60] Schmittgen T.D., Livak K.J. (2008). Analyzing real-time PCR data by the comparative C(T) method. Nat. Protoc..

[61] Lanubile A., Logrieco A., Battilani P., Proctor R.H., Marocco A. (2013). Transcriptional changes in developing maize kernels in response to fumonisin-producing and nonproducing strains of *Fusarium verticillioides*. Plant Sci..

[62] Rocha L.O., Barroso V.M., Andrade L.J., Pereira G.H.A., Ferreira-Castro F.L., Duarte A.P., Michelotto M.D., Correa B. (2016). *FUM* gene expression profile and fumonisin production by *Fusarium verticillioides* inoculated in *Bt* and non-*Bt* maize. Front. Microbiol..

[63] Bertuzzi T., Rastelli S., Mulazzi A., Pietri A. (2012). Evaluation and improvement of extraction methods for the analysis of aflatoxins B1, B2, G1 and G2 from naturally contaminated maize. Food Anal. Methods.

[64] Pietri A., Bertuzzi T. (2012). Simple phosphate buffer extraction for the determination of fumonisins in masa, maize and derived products. Food Anal. Methods.

[65] Clewer A.G., Scarisbrick D.H. (2001). Practical Statistics and Experimental Design for Plant and Crop Science.

