# Supplementary material for: Fusarium verticillioides and Aspergillus flavus Co-Occurrence Influences Plant and Fungal Transcriptional Profiles in Maize Kernels and In Vitro
Source: Toxins (Basel). 2021 Sep 24;13(10):680. doi: 10.3390/toxins13100680 (PMC8537323; doi:10.3390/toxins13100680)
Supplement: Supplementary file 1 [file toxins-13-00680-s001.zip › Supplementary_materials.pdf]

Supplementary Materials: *Fusarium verticillioides* and *Aspergillus flavus* Co-Occurrence Influences Plant and Fungal Transcriptional Profiles in Maize Kernels and *in Vitro*

Alessandra Lanubile, Paola Giorni, Terenzio Bertuzzi, Adriano Marocco and Paola Battilani

Table S1: Analysis of variance (ANOVA) of aflatoxin B1 (AFB1) contamination in maize kernels after 21 days of incubation in the different theses considered (mock, inoculation with *Aspergillus flavus* (*AF*), inoculation with *A. flavus* and *Fusarium verticillioides* (*AF+FV*) at three different temperatures (20, 25 and 30 °C). Data refer to mean data; all experiments were conducted with three replicates. *$P \leq 0.05$; ** $P \leq 0.01$.

**Kernel assay**	***AFB1***	
THESIS	**	
MOCK	0.5	C
AF	96.8	A
AF+FV	3.3	B
TEMPERATURE	n.s.	
20°C	109.7	
25°C	11.7	
30°C	7.4	

Table S2: Analysis of variance (ANOVA) of fumonisins B1+B2 (FBs) and aflatoxin B1 (AFB1) contamination in artificial liquid medium (PDB) after 21 days of incubation in the different theses considered (inoculation with *Fusarium verticillioides* (*FV*), inoculation with *Aspergillus flavus* (*AF*), inoculation with *A. flavus* and *F. verticillioides* (*AF+FV*) at three different temperatures (20, 25 and 30 °C). *$P \leq 0.05$; ** $P \leq 0.01$.

***In vitro* assay**	***FBS***		***AFB1***	
THESIS	**			
FV	26424	A		
AF+FV	2665	B		
THESIS			*	
AF			56171	A
AF+FV			12130	B
TEMPERATURE	*		n.s.	
20°C	19144	A	23367	
25°C	18663	AB	44403	
30°C	5823	B	34684	
